# Hamstring Anterior Cruciate Ligament Autograft Contributes to a Delayed Symptomatic Cyclops Lesion: A Case Report

**DOI:** 10.7759/cureus.56529

**Published:** 2024-03-20

**Authors:** Matthew P Corsi, Hussein F Darwiche, Fong Nham, Tannor Court, Henry Goitz

**Affiliations:** 1 School of Medicine, Wayne State University, Detroit, USA; 2 Department of Orthopaedic Surgery, Detroit Medical Center, Detroit, USA

**Keywords:** hamstring autograft, sutures, unraveling, delayed, cyclops, acl reconstruction

## Abstract

Cyclops lesions are characterized as fibroid nodules with granulation tissue that looks similar to a cyclops eye during arthroscopy. These are rare postoperative complications following anterior cruciate ligament reconstruction (ACLR), presenting typically within six months of their reconstruction. This case report presents a 21-year-old male, three years following hamstring autograft ACLR, with a symptomatic cyclops lesion. Contrary to the reported literature, this delayed presentation showed a painful flexion contracture of the knee and intraoperative findings consistent with a cyclops lesion. The treatment consisted of surgical debridement and notchplasty with subsequent posterior medial and lateral meniscal horn repairs. This case report presents a lesson to indicate that cyclops lesions can occur in a delayed setting following ACLR and to show a technique for successful surgical management of the lesion.

## Introduction

An anterior cruciate ligament (ACL) tear is a common sports-related injury most frequently occurring in the non-contact setting of a planted foot and sudden change in direction or speed, with peak incidence occurring between ages 19-25 in males and 14-18 in females [1]. In the majority of cases, the treatment of these injuries consists of anterior cruciate ligament reconstruction (ACLR) aimed at restoring function and knee joint stability in the sagittal and axial planes. The optimal graft choice remains a debated topic in the literature, with multiple options for graft types and sources. Including both allograft and autograft, with the patellar tendon (bone-tendon-bone), quadricep tendon, and hamstring tendon as the main source for autografts. Despite the continued debate, there exists a consensus in the literature pointing towards bone-tendon-bone autograft as the “gold standard” especially in the high-demand athletic patient population, and mainly due to primary bone-to-bone healing and subsequent accelerated graft incorporation [2]. Although gaining recent traction, the quadriceps tendon autograft remains the least used graft, owed to the technically demanding harvesting process. However, it has been shown to yield consistent and replicable grafts and avoids most morbidities associated with the other autograft options [3]. Hamstring tendon autograft has been noted to have the highest tensile graft strength as demonstrated by some biomechanical studies; however, this benefit comes at the expense of postoperative weakness in knee flexion and higher infection rates [4,5]. Allograft options provide the benefit of eliminating donor site morbidity, faster operating time, and better cosmesis; however, they are at the risk of higher graft-related failures and hence are reserved for active but less demanding patients [2].

Despite the relatively high success rates, complications such as re-rupture, continued instability, infection, cyclops lesion development, and others can occur postoperatively. Cyclops lesions, a term coined by Jackson and Schaefer in 1990, constitute a fibrous nodule and granulation tissue with an appearance comparable to a cyclops eye with overlying vascularity when viewed arthroscopically [6]. Although cyclops lesions have been reported in patients in the absence of ACL reconstructions [7], ACL reconstruction is considered a significant risk factor for their development. Literature has shown that these lesions demonstrate an incidence ranging between 1.9%-10.9% [8], with 93% of cases being usually diagnosed within six months of initial surgery [9]. Symptomatic lesions are termed cyclops syndrome when the patient exhibits loss of knee extension with potentially audible mechanical locking and pain at terminal extension [10]. In general, the loss of 20 degrees of extension progresses on average of four months post-ACLR with complete resolution of symptoms following lesion excision [11]. In this report, we describe a case of a 21-year-old male presenting three years following hamstring autograft ACLR with a symptomatic cyclops lesion seen contiguous with a partial unraveling of the hamstring autograft.

## Case presentation

**Figure 1:**
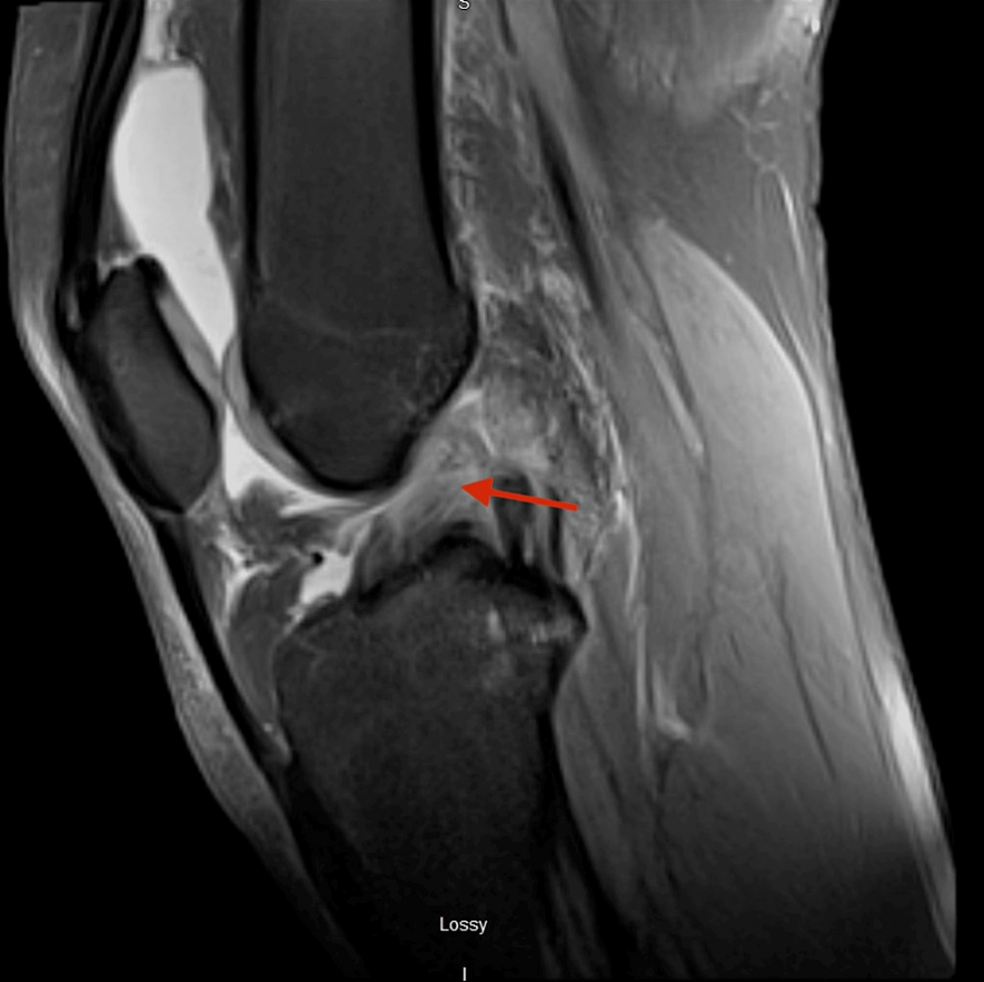
T2 sagittal magnetic resonance imaging of the left knee The image shows the preoperative anterior cruciate rupture (red arrow).

**Figure 2:**
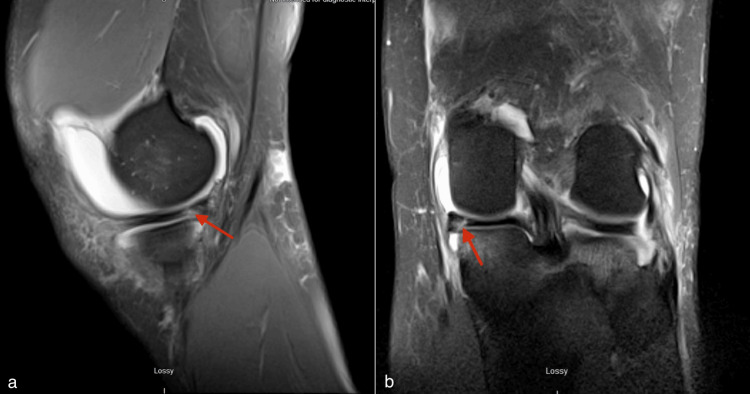
Preoperative MRI image of the left knee a) T2 sagittal magnetic resonance imagining of the preoperative left knee demonstrating the vertical medial meniscus tear (red arrow). b) T2 coronal magnetic resonance imagining of the preoperative left knee with a vertical medial meniscus tear (red arrow).

A 21-year-old highly active male with no significant past medical history initially presented to an outside surgeon three weeks after a twisting injury to the left knee with significant swelling. Patient records were available for this review. The patient localized the pain to the posterior knee with some restriction of knee motion. The patient exhibited a positive Lachman, and an MRI demonstrated a full-thickness ACL tear and a vertical tear of the peripheral medial meniscus (Figures 1, 2). Surgery was scheduled nearly six weeks post-injury. Hamstring autograft utilizing gracilis and semitendinosus was harvested and tripled bundled measuring 8 mm on the femoral side and 9 mm on the tibial side. All-inside knotless implants were used to repair the meniscus. A post-operative hinged knee brace was placed with instructions for toe touch weight bearing for the first month followed by progressive weight bearing over three weeks. The patient was limited to a range of motion 0-90 for the first month, with a hinged knee brace on at all times, and physical therapy for two months. The first postoperative follow-up was one month during which the patient was instructed to initiate knee flexion beyond 90 degrees and 50% weight bearing until 6 weeks. The second postoperative follow-up was at eight weeks where knee range of motion was recorded as 2-120 degrees and full return to activities at the third follow-up at nine months after surgery.

**Figure 3:**
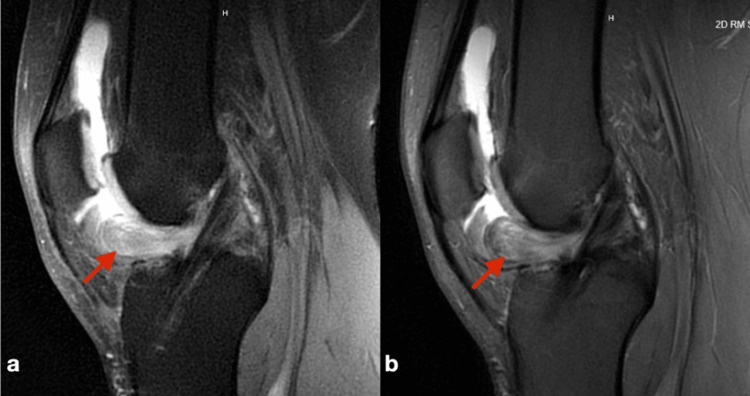
MRI images revealing the cyclops lesion a) T1 magnetic resonance imaging sagittal view of the left knee demonstrating the cyclops lesion (red arrow) anterior to the anterior cruciate ligament reconstruction. b) T2 magnetic resonance imagining of the same sagittal view with cyclops lesion (red arrow).

**Figure 4:**
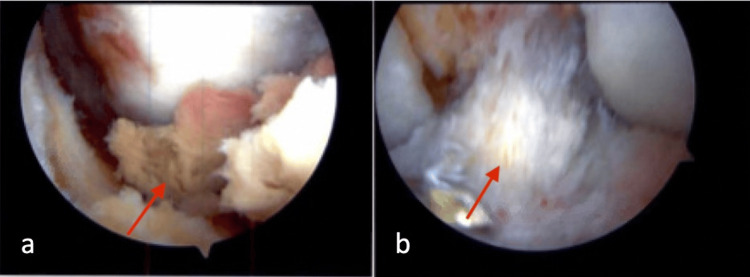
Arthroscopic visualization before and after the treatment a) Arthroscopic visualization of the cyclops lesions (red arrow). b) Arthroscopic visualization of anterior cruciate ligament (red arrow) after cyclops debridement.

The patient presented to our office three years post primary ACLR with complaints of the knee “giving out,” locking and catching. The patient exhibited a flexion contracture with a passive range of motion of 7-120 degrees; he limped with ambulation. Lachman's test was negative. Afterward, an MRI was subsequently obtained, and it demonstrated a cyst lesion anteriorly and contiguous to the ACLR (Figures 3a, 3b). An arthroscopic excision was scheduled. Intraoperatively, the lesion was communicating with the ACL graft and measured 2.5 x 1.5 x 0.7 cm (Figure 4). Pathology reported the specimen as “tendon tissue with degenerative changes and necrosis.” Additionally, there was an unraveling of suture that suggested this cyclops to be secondary, at least in part, to the unraveling of a portion of the autograft bundle. Notchplasty was performed to assist in achieving full knee extension. The posterior horns of both the medial and lateral meniscus were noted to be torn and unstable. An all-inside meniscal repair was performed on both menisci. Full knee extension was achieved with subsequent manipulation under anesthesia. One year post-operatively, the patient remains asymptomatic with full knee extension and pain-free.

## Discussion

The symptomatology of a cyclops lesion three years post-ACLR, with the lesion being caused, at least in part, by a free-floating strand of hamstring autograft in the joint originating from the unraveling of the graft presents a unique cause for a cyclops syndrome. Studies suggest that the classic cyclops lesion is a result of a hypertrophied or disorganized mass of graft tissue secondary to the anterior placement of the tibial tunnels [12]. Other explanations include nodules from the native ACL bundle immediately after complete or partial tears with avulsions of the femur [13] and the tibia [14] or repeated microtrauma to the joint with a subsequent inflammatory process leading to the formation of the lesion; the incidence and diagnosis of a cyclops lesion was reported at 93% within six months of surgery [15]. Contrary to these studies, the patient in this report presented three years after hamstring autograft ACLR with the “typical” presentation of a flexion contracture with knee extension pain with an intraoperatively identifiable cyclops that blocked full knee extension and incorporated a portion of the ACL hamstring graft.

In more recent years there have been questions as to whether particular graft selection leads to a higher incidence of cyclops lesions and syndrome following ACLR. Studies have demonstrated that bone patellar bone autograft compared to hamstring autograft as well as female sex and increased BMI leads to increased rates of cyclops syndrome [16]. Quadriceps tendon autograft is becoming a popular choice of graft selection among surgeons as it shows similar biomechanical outcomes as well as improved cosmesis in comparison to other graft selections. However, studies have demonstrated that cyclops syndrome after double-bundle and quadriceps graft ACLR is greater than in single-bundle and hamstring graft ACLR [9]. However, these results are likely due to the increased graft size and greater volume of graft that impinges on the PCL ligament synovium posteriorly, causing a lesion that arises from the synovium of the posterior cruciate ligament rather than the graft [9]. With that being said further research has shown that male patients with a femoral tunnel greater than 9.25 mm or greater increase the rate of cyclops lesions. As a result, the authors stress implanting a graft size of 9.00 mm with an emphasis on achieving early terminal extension to greatly reduce the risk for arthrofibrosis in the utilization of a quadriceps tendon autograft [17]. 

In the efforts to reduce the incidence of cyclops lesions, Nagira et al. demonstrate the debridement of the bone in and around the bone tunnel prior to the placement of the double bundle hamstring graft. The study showed that those with bone debridement showed a lower prevalence of cyclops lesions on post-operative MRI than the non-debrided, which was statistically significant. This lower prevalence also stood true for those with symptomatic lesions [14]. Literature has demonstrated that patients do exceptionally well following the resection of a cyclops lesion. If a symptomatic cyclops lesion does develop, studies have shown that following resection of the lesion patients can return to their previous activities and have minimal to no pain at terminal extension [18]. Furthermore, new techniques have been developed within the last two years to excise symptomatic cyclops lesions using a minimally invasive approach utilizing the NanoScope. The goal is to reduce postoperative swelling and pain in comparison to using the conventional arthroscopic route [19].

This case presents the importance of considering a cyclops lesion with loss of terminal extension numerous years following ACLR. It also presents an unusual development of the lesion, in both time and etiology. While the development of a cyclops lesion is rare and typically presents earlier in the rehabilitation course it is still an important differential diagnosis to have in the workup of postoperative stiffness following ACLR. Diagnosis of a cyclops lesion involves careful evaluation of the patient through meticulous history and physical exam, radiographic imaging, and MRI. Arthroscopic resection of the lesion is warranted if symptomatic which has demonstrated great results and is becoming more minimally invasive through new technologies.

## Conclusions

This 21-year-old patient presented with a cyclops lesion three years after hamstring autograft ACLR with a flexion contracture and knee pain with forced knee extension. Arthroscopically, the cyclops lesion was found to block full knee extension and be continuous in origin to an unraveled portion of a hamstring autograft. The treatment consisted of surgical debridement and notchplasty with subsequent posterior medial and lateral meniscal horn repairs. We are unaware of this unique pathophysiology to be previously described in the literature.
